# Association Between Periodontal Health and Perioperative Glycemic Outcomes in Diabetic Patients Undergoing Dental Procedures Under General Anesthesia: A Retrospective Cohort Study

**DOI:** 10.7759/cureus.115668

**Published:** 2026-09-02

**Authors:** Pritha Negi, Tulasi R Jangala, Tanveer Karpe, Deeksha Kallala, Anupama Betigeri, Maitri Vipulkumar Doshi

**Affiliations:** 1 Department of Prosthodontics, Crown and Bridge, K. M. Shah Dental College and Hospital, Sumandeep Vidyapeeth (Deemed to be University), Vadodara, IND; 2 Department of Anaesthesia and Critical Care, Seventh Day Adventist Hospital, Aizawl, IND; 3 Department of Oral and Maxillofacial Surgery, Faculty of Dentistry, Taif University, Taif, SAU; 4 Department of Public Health, Sixteenth Street Community Health Centers, Milwaukee, USA; 5 Department of Physiology, Manav Rachna Dental College, Faridabad, IND; 6 Department of Medicine, Faculty of Medicine, University Geomedi, Tbilisi, GEO

**Keywords:** diabetes mellitus, general anesthesia, periodontitis, perioperative glycemic control, postoperative complications

## Abstract

Introduction

Periodontitis and diabetes are closely related, but the influence of periodontal disease severity on perioperative glycemic outcomes during dental procedures under general anesthesia remains unclear. This study evaluated the association between periodontal disease severity and perioperative glycemic outcomes in diabetic patients.

Materials and methods

This retrospective study included 72 patients with diabetes who underwent elective dental procedures under general anesthesia between January 2015 and December 2018. The patients were categorized into mild periodontitis (n = 43) and severe periodontitis (n = 29) groups. Demographic, diabetic, periodontal, anesthetic, and perioperative data were retrieved from the electronic medical records. Periodontal parameters included plaque index, bleeding on probing, probing pocket depth, clinical attachment loss, and the number of remaining teeth. Perioperative glycemic outcomes were assessed using preoperative, intraoperative, and postoperative blood glucose levels; glycemic variability; and the incidence of hyperglycemia and hypoglycemia. Postoperative complications were also assessed. Statistical analysis was then performed.

Results

Patients with severe periodontitis had significantly higher glycated hemoglobin (HbA1c) levels than those with mild disease (8.9 ± 1.3% vs. 7.6 ± 1.2%; p < 0.001). Mean preoperative (185.6 ± 42.4 vs. 142.3 ± 38.1 mg/dL), intraoperative (210.8 ± 48.2 vs. 155.4 ± 41.5 mg/dL), postoperative (225.1 ± 52.3 vs. 161.2 ± 45.8 mg/dL), and mean perioperative blood glucose levels (207.2 ± 43.5 vs. 153.0 ± 38.2 mg/dL) were significantly higher in patients with severe periodontitis (all p < 0.001). Glycemic variability was also significantly greater (41.3 ± 14.2 vs. 28.5 ± 10.1; p < 0.001). Perioperative hyperglycemia was observed in 22 (75.9%) patients with severe periodontitis compared with 12 (27.9%) patients with mild periodontitis (p < 0.001). Surgical site infection occurred in 9 (31.0%) and 3 (7.0%) patients (p = 0.014), delayed wound healing in 11 (37.9%) and 4 (9.3%) patients (p = 0.006), and hospital stay exceeding 24 hours in 15 (51.7%) and 8 (18.6%) patients (p = 0.004), respectively. The periodontal destruction and perioperative glycemic parameters showed a significant positive correlation (r = 0.58-0.67; all p < 0.001).

Conclusion

Severe periodontitis was associated with poorer perioperative glycemic control, greater glycemic variability, and more frequent postoperative complications in diabetic patients undergoing dental procedures under general anesthesia. These findings indicate an association between periodontal disease severity and perioperative outcomes but do not establish a causal relationship. Prospective studies are needed to determine whether periodontal management can improve perioperative outcomes.

## Introduction

Persistent hyperglycemia brought on by decreased insulin secretion, action, or both is a hallmark of diabetes mellitus (DM), a chronic metabolic disease. Despite significant advances in perioperative diabetic care, maintaining stable blood glucose levels during surgical procedures remains a major clinical challenge [1]. Patients with diabetes undergoing dental procedures under general anesthesia are particularly vulnerable to fluctuations in blood glucose levels caused by surgical stress, anesthetic agents, inflammatory responses, and perioperative fasting [2]. Poor glycemic control during the perioperative period has been associated with an increased risk of postoperative infection, delayed wound healing, prolonged hospitalization, and other adverse clinical outcomes [2,3].

Currently acknowledged as the sixth major complication of diabetes, periodontal disease is one of the most common chronic inflammatory disorders in the world. The relationship between diabetes and periodontitis is bidirectional [4]. Chronic hyperglycemia impairs host immune function, alters collagen metabolism, and compromises tissue repair, thereby increasing the susceptibility to periodontal destruction [5]. Conversely, periodontal inflammation contributes to the systemic inflammatory burden through the release of pro-inflammatory cytokines, which promote insulin resistance and further impair glycemic control [6]. Consequently, patients with severe periodontitis often exhibit higher glycated hemoglobin (HbA1c) levels and poorer metabolic control than those with healthy periodontal tissues [7].

Although several studies have demonstrated that periodontal therapy can improve long-term glycemic control, considerably less attention has been directed toward the influence of periodontal health on immediate perioperative glucose regulation [4-7]. The perioperative period represents a physiologically stressful state in which pre-existing systemic inflammation may amplify metabolic disturbances, potentially increasing glucose variability and the incidence of perioperative hyperglycemia [3]. Understanding whether periodontal disease severity influences perioperative glycemic outcomes could provide clinicians with an additional modifiable risk factor to optimize before elective dental procedures under general anesthesia.

Furthermore, identifying an association between periodontal status and perioperative metabolic control may facilitate better interdisciplinary collaboration between dentists, periodontists, anesthesiologists, oral surgeons, and physicians involved in the management of patients with diabetes. Such evidence could support the incorporation of periodontal assessment into routine preoperative evaluations and contribute to improved surgical safety and postoperative recovery. The purpose of this study was to assess the relationship between perioperative glycemic results and periodontal health in diabetic individuals receiving general anesthesia for dental procedures.

The primary objective of this study was to evaluate the association between periodontal disease severity and perioperative glycemic control in diabetic patients undergoing dental procedures under general anesthesia. The secondary objectives were to compare glycemic variability and the incidence of perioperative hyperglycemia and hypoglycemia between periodontal disease severity groups, evaluate the association between periodontal disease severity and postoperative complications, and assess correlations between periodontal parameters and perioperative glycemic measures.

## Materials and methods

Study design, setting, and ethical approval

The Department of Physiology at Manav Rachna Dental College in Faridabad, India, worked with the Department of Oral and Maxillofacial Surgery to perform this retrospective observational cohort study. The electronic medical records, anesthesia records, laboratory data, and periodontal charts of eligible diabetic patients who underwent elective dentoalveolar surgical procedures under general anesthesia between January 2015 and December 2018 were retrospectively reviewed. Prior to commencement, ethical approval was obtained from the Institutional Ethics Committee. The Ethics Committee waived the need for written informed permission because the study comprised retrospective analysis of anonymised patient records without direct patient involvement (MRIIRS/MRDC/SDS/IEC/2019/128). Patient confidentiality was maintained throughout the study by assigning unique study identification numbers and removing all personal identifiers prior to data analysis.

Sample size estimation

The sample size was estimated a priori using G*Power software version 3.1.9.7 (Heinrich Heine University Düsseldorf, Düsseldorf, Germany). Based on the anticipated between-group difference in perioperative blood glucose levels between patients with mild and severe periodontal disease, an independent-samples t-test was used for the sample size estimation, with a two-sided significance level (α) of 0.05, 80% statistical power, and an anticipated moderate-to-large effect size (Cohen's d = 0.65) [8]. The estimated sample size was 76 participants (38 per group). The final study cohort comprised 72 eligible patients with complete records available for analysis.

The independent-samples t-test was selected for the a priori sample size estimation because the primary objective was to compare perioperative glycemic control between the two predefined periodontal disease severity groups. Although blood glucose was recorded repeatedly at different perioperative time points, the repeated measurements were subsequently analyzed using a linear mixed-effects model to account for within-patient correlation and to evaluate the effects of periodontal disease severity, time, and their interaction. Thus, the sample size calculation was based on the planned primary between-group comparison, whereas the final statistical analysis appropriately accounted for the longitudinal structure of the repeated glucose measurements.

Participant selection

The hospital records were screened consecutively to identify eligible participants. To reduce the potential confounding effects of procedural variability on perioperative glycemic outcomes, only patients who underwent the same category of elective dentoalveolar surgical procedures under general anesthesia were included in the study. These procedures primarily comprised multiple tooth extractions, surgical removal of impacted teeth, and other comparable dentoalveolar surgeries requiring similar operative techniques and anesthetic management. Patients undergoing major oral and maxillofacial procedures, including orthognathic surgery, maxillofacial trauma surgery, oncologic resections, or reconstructive procedures, were excluded to ensure a relatively homogeneous surgical cohort and minimize differences in surgical stress response and perioperative metabolic alterations.

Patients were required to have undergone elective dental procedures under general anesthesia, have complete anesthesia and perioperative blood glucose records, have a comprehensive periodontal examination documented within three months before surgery, and have an HbA1c measurement available within the same three-month preoperative period. The three-month window was predefined to ensure that periodontal and metabolic assessments were reasonably proximate to the surgical event while allowing inclusion of eligible patients in this retrospective record-based study. Table 1 summarizes the eligibility of study participants.

**Table 1:** Inclusion and exclusion criteria for study participants.

Inclusion criteria	Exclusion criteria
Adult patients aged ≥ 18 years.	Incomplete medical or periodontal records.
Physician-confirmed diagnosis of Type 1 or Type 2 diabetes mellitus.	Pregnancy.
Receiving continuous subcutaneous insulin therapy for ≥ 3 months prior to surgery.	Systemic disorders (Immunodeficiency, renal disorders).
Underwent elective dental procedures under general anesthesia.	Chronic corticosteroid therapy or Chemotherapy
Complete anesthesia and perioperative blood glucose records available.	Receiving insulin pump therapy or inhaled insulin.
Comprehensive periodontal examination performed within 3 months before surgery.	Active oral infections unrelated to periodontal disease.
Documented HbA1c value available within 3 months prior to the procedure.	Fewer than three documented perioperative blood glucose measurements.

Assessment of periodontal health

Periodontal status was obtained from standardized periodontal examination records completed by calibrated periodontists before the surgical procedure. The periodontal examination included plaque index (PI), bleeding on probing (BOP), probing pocket depth (PPD), clinical attachment level (CAL), and the number of remaining teeth [9]. Periodontal disease was classified according to the 2017 World Workshop classification of periodontal and peri-implant diseases and conditions [10]. Participants were initially categorized as having periodontal health/gingivitis, Stage I-II periodontitis, or Stage III-IV periodontitis.

For the primary analysis, these categories were subsequently dichotomized into a mild periodontal disease group (periodontal health/gingivitis and Stage I-II periodontitis) and a severe periodontal disease group (Stage III-IV periodontitis). This analytical grouping was prespecified to provide a clinically interpretable comparison between participants with no or limited periodontal destruction and those with advanced periodontitis. The 2017/2018 classification distinguishes gingival health/gingivitis from periodontitis and further stratifies periodontitis according to severity and complexity; Stage I and II represent the earlier stages of periodontitis, whereas Stage III and IV represent advanced disease with greater periodontal destruction and complexity [10]. Thus, the grouping used in the present study represents an analytical categorization based on severity rather than a modification of the established periodontal classification.

Data collection

Demographic, medical, periodontal, anesthetic, and perioperative data were extracted. Demographic characteristics included age, sex, body mass index (BMI), smoking status, alcohol use, and American Society of Anesthesiologists (ASA) physical state categorization [11]. Diabetes-related variables included type of diabetes, duration of diabetes, duration of insulin therapy, daily insulin dose, concurrent antidiabetic medications, and HbA1c levels. Perioperative variables included the type and duration of dental procedure, duration of anesthesia, airway management technique, estimated blood loss, intraoperative fluid administration, corticosteroid administration, and postoperative recovery characteristics. Blood glucose measurements recorded preoperatively, immediately after anesthesia induction, at hourly intraoperative intervals, at the completion of surgery, in the post-anesthesia recovery unit, and during the first 24 postoperative hours were retrieved from anesthesia records and hospital laboratory databases.

For retrospective data extraction, blood glucose measurements were classified according to their documented timing relative to anesthesia induction and surgery. The preoperative value was defined as the glucose measurement obtained closest to induction of general anesthesia before induction. The intraoperative value was defined as the first glucose measurement documented after induction of anesthesia and before completion of surgery. The postoperative value was defined as the first glucose measurement documented after completion of surgery. The exact time interval from anesthesia induction or surgical completion to each glucose measurement was recorded. Where multiple measurements were available within a defined period, the measurement closest to the prespecified time point was selected.

Outcome measures

The primary outcome of the study was perioperative glycemic stability. This was assessed using preoperative, intraoperative, and postoperative blood glucose concentrations; mean perioperative blood glucose levels; glucose variability expressed as standard deviation and coefficient of variation; and the incidence of perioperative hyperglycemia (>180 mg/dL) and hypoglycemia (<70 mg/dL). Secondary outcomes included postoperative surgical site infection, delayed wound healing, postoperative bleeding requiring intervention, and duration of hospital stay.

Calibration and quality assurance

Data extraction of surgical records was independently performed by two calibrated investigators (TK, TRJ). Prior to the commencement of data collection, both investigators performed calibration exercises to ensure consistency in the interpretation of clinical records. Ten percent of randomly selected patient records were re-evaluated after a two-week interval to determine intraobserver and interobserver reliability. Reliability for continuous variables was assessed using the ICC, whereas that for categorical variables was evaluated using Cohen's kappa coefficient. Agreement values greater than 0.80 were considered indicative of excellent reliability. The intraobserver ICC value was 0.93 (95% CI: 0.89-0.96), and the interobserver ICC value was 0.91 (95% CI: 0.87-0.95). Cohen's kappa coefficients were 0.89 for intraobserver agreement and 0.87 for interobserver agreement, indicating excellent reproducibility and consistency in data extraction.

Control of bias

Several methodological strategies have been implemented to minimize potential bias. Consecutive sampling reduced selection bias, and predefined inclusion and exclusion criteria ensured a homogeneous study population. Standardized periodontal examination protocols and electronic anesthesia records minimized measurement bias. Investigators responsible for postoperative outcome assessment were blinded to the periodontal disease classification whenever feasible. Potential confounding variables, including age, sex, BMI, smoking status, HbA1c, duration of diabetes, ASA physical status, duration of surgery, and corticosteroid administration, were adjusted during multivariable statistical analyses. Missing data were evaluated systematically, and multiple imputation techniques were applied when the proportion of missing data was less than 10%. Records with substantial missing information were excluded from the final analysis. Sensitivity analysis was performed to assess the robustness of the findings.

Statistical analysis

A statistician (PN) used IBM SPSS Statistics version 25.0 (IBM Corp., Armonk, NY, USA) to run statistical analyses on coded data. The Shapiro-Wilk test and Q-Q plot analysis were used to determine whether continuous variables were normal. Non-normally distributed data were displayed as median and interquartile range (IQR), whereas regularly distributed variables were summarized as mean ± standard deviation (SD). Categorical variables were expressed as frequencies and percentages. Baseline demographic and clinical characteristics among periodontal disease groups were compared using independent t-test for normally distributed continuous variables, the Mann-Whitney U test for non-normally distributed variables, and the chi-square or Fisher's exact test for categorical variables, as appropriate. Changes in perioperative blood glucose levels across multiple time points were analyzed using a linear mixed-effects model with patient-specific random intercepts to account for repeated measurements. Fixed effects included periodontal disease severity, time, and the interaction between periodontal disease severity and time. Post hoc analyses were conducted using Bonferroni-adjusted pairwise comparisons. Statistical significance was defined as two-sided p-values less than 0.05.

## Results

A total of 72 diabetic patients who underwent dental procedures under general anesthesia fulfilled the eligibility criteria and were included in the final analysis. Based on periodontal disease severity, 43 patients (59.7%) were categorized as having mild periodontitis, whereas 29 patients (40.3%) had severe periodontitis. No statistically significant differences were observed between the mild and severe periodontitis groups regarding age (p = 0.204), sex distribution (p = 0.359), BMI (p = 0.276), diabetes type (p = 0.297), duration of diabetes (p = 0.294), duration of insulin therapy (p = 0.481), daily insulin dose (p = 0.064), or duration of surgery (p = 0.336). However, patients with severe periodontitis demonstrated significantly higher HbA1c levels (8.9 ± 1.3% vs. 7.6 ± 1.2%; p < 0.001), a significantly greater proportion of smokers (48.3% vs. 23.3%; p = 0.027), and a higher frequency of ASA physical status III/IV (p = 0.041), indicating poorer baseline metabolic control and greater systemic disease burden among patients with severe periodontal disease (Table 2).

**Table 2:** Baseline demographic, diabetic, and perioperative characteristics of the study participants. Data are presented as mean ± standard deviation (SD) for normally distributed continuous variables and n (%) for categorical variables. Intergroup comparisons were performed using the independent samples t-test for continuous variables and the Chi-square (χ²) test for categorical variables, p-values are two-tailed. *p < 0.05 was considered statistically significant. ASA: American Society of Anesthesiologists, BMI: body mass index, HbA1c: glycated hemoglobin.

Variable	Overall (n = 72)	Mild periodontitis (n = 43)	Severe periodontitis (n = 29)	Test statistic	p-value
Age (years), Mean ± SD	58.2 ± 11.5	56.8 ± 12.1	60.3 ± 10.4	t = -1.28	0.204
Male, n (%)	40 (55.6%)	22 (51.2%)	18 (62.1%)	χ² = 0.84	0.359
Female, n (%)	32 (44.4%)	21 (48.8%)	11 (37.9%)		
BMI (kg/m²), Mean ± SD	29.4 ± 4.2	29.0 ± 4.5	30.1 ± 3.7	t = -1.10	0.276
Type 1 diabetes, n (%)	11 (15.3%)	5 (11.6%)	6 (20.7%)	χ² = 1.09	0.297
Duration of diabetes (years), Mean ± SD	12.5 ± 6.8	11.8 ± 6.5	13.5 ± 7.2	t = -1.06	0.294
HbA1c (%), Mean ± SD	8.1 ± 1.4	7.6 ± 1.2	8.9 ± 1.3	t = -4.34	< 0.001*
Duration of insulin therapy (years)	6.8 ± 4.1	6.5 ± 4.3	7.2 ± 3.8	t = -0.71	0.481
Daily insulin dose (Units/day)	62.5 ± 24.1	58.2 ± 22.5	68.9 ± 25.2	t = -1.88	0.064
Smoking, n (%)	24 (33.3%)	10 (23.3%)	14 (48.3%)	χ² = 4.89	0.027*
ASA III/IV, n (%)	27 (37.5%)	12 (27.9%)	15 (51.7%)	χ² = 4.19	0.041*
Duration of surgery (minutes)	128.5 ± 45.2	124.3 ± 42.8	134.8 ± 48.1	t = -0.97	0.336

As expected, patients with severe periodontitis exhibited significantly greater periodontal destruction than those with mild periodontitis did. PI (2.4 ± 0.7 vs. 1.2 ± 0.6; p < 0.001) and BOP (52.8 ± 18.5% vs. 18.5 ± 10.2%; p < 0.001) were markedly higher in the severe periodontitis group. Likewise, mean PPD (5.8 ± 1.4 mm vs. 2.9 ± 0.8 mm; p < 0.001) and CAL (6.5 ± 1.7 mm vs. 3.2 ± 1.1 mm; p < 0.001) were significantly greater among patients with severe disease. In contrast, the number of remaining teeth was significantly lower in the severe periodontitis group (17.1 ± 4.5 vs. 24.5 ± 3.2; p < 0.001). All periodontal variables demonstrated large effect sizes, confirming a clear distinction between the two disease categories (Table 3).

**Table 3:** Comparison of periodontal clinical parameters between mild and severe periodontitis groups. Data are presented as mean ± standard deviation (SD) and median (interquartile range [IQR]). Comparisons between groups were performed using the Mann–Whitney U test. Effect size is expressed as correlation coefficient (r). *p < 0.05 was considered statistically significant. PPD: probing pocket depth.

Parameter	Mild periodontitis (n = 43)		Severe periodontitis (n = 29)		Test statistic (U)	p-value	Effect size (r)
	Mean ± SD	Median (Q1-Q3)	Mean ± SD	Median (Q1-Q3)			
Plaque Index	1.2 ± 0.6	1.1 (0.8-1.5)	2.4 ± 0.7	2.3 (1.9-2.8)	178.5	< 0.001*	0.68
Bleeding on probing (%)	18.5 ± 10.2	16.0 (10.0-25.0)	52.8 ± 18.5	55.0 (38.0-67.0)	104.0	< 0.001*	0.78
Mean probing pocket depth (mm)	2.9 ± 0.8	2.8 (2.4-3.3)	5.8 ± 1.4	5.7 (4.7-6.9)	50.5	< 0.001*	0.86
Clinical attachment loss (mm)	3.2 ± 1.1	3.0 (2.5-3.8)	6.5 ± 1.7	6.2 (5.1-7.8)	42.0	< 0.001*	0.88
Number of remaining teeth	24.5 ± 3.2	25.0 (23.0-27.0)	17.1 ± 4.5	17.0 (14.0-20.0)	131.5	< 0.001*	0.74

Patients with severe periodontitis experienced significantly poorer glycemic control during the perioperative period. Mean preoperative blood glucose levels were significantly higher in the severe periodontitis group (185.6 ± 42.4 mg/dL) than in the mild periodontitis group (142.3 ± 38.1 mg/dL; p < 0.001). Similar findings were observed for intraoperative blood glucose (210.8 ± 48.2 mg/dL vs. 155.4 ± 41.5 mg/dL; p < 0.001) and postoperative blood glucose concentrations (225.1 ± 52.3 mg/dL vs. 161.2 ± 45.8 mg/dL; p < 0.001). The mean perioperative glucose level was also significantly higher in patients with severe periodontitis (207.2 ± 43.5 mg/dL) than in those with mild disease (153.0 ± 38.2 mg/dL; p < 0.001). Furthermore, glycemic variability was significantly greater in the severe periodontitis group (41.3 ± 14.2 vs. 28.5 ± 10.1; p < 0.001), as shown in Table 4.

**Table 4:** Comparison of perioperative glycemic outcomes according to periodontal disease severity. Continuous variables are presented as mean ± standard deviation (SD) and median (interquartile range [IQR]). Continuous variables were compared using the independent-samples t-test. *p < 0.05 was considered statistically significant.

Variable	Mild periodontitis (n = 43)		Severe periodontitis (n = 29)		Test statistic	p-value	Effect size (Cohen's d)
	Mean±SD	Median (IQR)	Mean±SD	Median (IQR)			
Preoperative glucose (mg/dL)	142.3 ± 38.1	138.0 (115.0-168.0)	185.6 ± 42.4	179.0 (154.0-212.0)	-4.44	< 0.001*	1.07
Intraoperative glucose (mg/dL)	155.4 ± 41.5	152.0 (122.0-185.0)	210.8 ± 48.2	205.0 (176.0-238.0)	-5.13	< 0.001*	1.24
Postoperative glucose (mg/dL)	161.2 ± 45.8	158.0 (126.0-192.0)	225.1 ± 52.3	218.0 (189.0-258.0)	-5.39	< 0.001*	1.31
Mean perioperative glucose (mg/dL)	153.0 ± 38.2	149.0 (121.0-182.0)	207.2 ± 43.5	200.0 (177.0-236.0)	-5.54	< 0.001*	1.33
Glycemic variability (SD)	28.5 ± 10.1	26.2 (20.8-35.1)	41.3 ± 14.2	39.5 (30.2-51.8)	-4.30	< 0.001*	1.05

Perioperative hyperglycemia (>180 mg/dL) occurred significantly more frequently in patients with severe periodontitis (p < 0.001), whereas the incidence of hypoglycemia (<70 mg/dL) did not differ significantly between the two groups (p = 0.362) (Table 5).

**Table 5:** Distribution of periodontitis according to glycemic level in study participants. Categorical data presented as frequency and percentage, Chi-square test applied to analyze the association between variables, and effect size presented as Cramer V.

Variable	Mild periodontitis (n = 43)	Severe periodontitis (n = 29)	Test statistic (χ²)	p-value	Effect size (Cramer V)
Hyperglycemia (>180 mg/dL), n (%)	12 (27.9%)	22 (75.9%)	16.22	< 0.001*	0.48
Hypoglycemia (<70 mg/dL), n (%)	2 (4.7%)	3 (10.3%)	0.83	0.362	0.1

Patients with severe periodontitis experienced significantly higher rates of adverse postoperative events than those with mild periodontitis did. Surgical site infections occurred more frequently in patients with severe periodontitis than in those with mild disease (OR = 5.97; 95% CI: 1.43-24.87; p = 0.014). Delayed wound healing was also significantly more common in the severe group (OR = 5.95; 95% CI: 1.65-21.42; p = 0.006). Although postoperative bleeding requiring intervention occurred more frequently in patients with severe disease, the difference was not statistically significant (p = 0.098). Additionally, patients with severe periodontitis had a significantly greater likelihood of requiring hospitalization for more than 24 h (OR = 4.69; 95% CI: 1.63-13.51; p = 0.004) (Table 6).

**Table 6:** Comparison of postoperative outcomes according to periodontal disease severity. Data are presented as n (%). Associations between periodontal disease severity and postoperative outcomes were evaluated using binary logistic regression. *p < 0.05 was considered statistically significant. CI: confidence interval; OR: odds ratio.

Outcome	Mild periodontitis (n = 43), n (%)	Severe periodontitis (n = 29), n (%)	Odds ratio	95% CI	p-value
Surgical site infection	3 (7.0%)	9 (31.0%)	5.97	1.43 – 24.87	0.014*
Delayed wound healing	4 (9.3%)	11 (37.9%)	5.95	1.65 – 21.42	0.006*
Postoperative bleeding	2 (4.7%)	5 (17.2%)	4.27	0.77 – 23.64	0.098
Hospital stay >24 hours	8 (18.6%)	15 (51.7%)	4.69	1.63 – 13.51	0.004*

Significant positive correlations were observed between the severity of periodontal disease and glycemic parameters. The mean PPD demonstrated a moderate positive correlation with HbA1c level (r = 0.58; p < 0.001) and mean perioperative blood glucose concentration (r = 0.63; p < 0.001). Similarly, clinical attachment loss was strongly and positively correlated with HbA1c (r = 0.62; p < 0.001) and postoperative blood glucose levels (r = 0.67; p < 0.001). These findings indicate that increased periodontal destruction was associated with progressively poorer perioperative glycemic control, reinforcing the potential influence of periodontal health on perioperative metabolic stability in diabetic patients undergoing dental procedures under general anesthesia (Table 7).

**Table 7:** Correlation between periodontal disease severity and glycemic parameters. Correlations were assessed using Pearson's correlation coefficient (r). *p < 0.05 was considered statistically significant. HbA1c: glycated hemoglobin.

Variable	Correlation coefficient (r)	p-value
Mean probing pocket depth vs. HbA1c	0.58	< 0.001*
Mean probing pocket depth vs. mean perioperative glucose	0.63	< 0.001*
Clinical attachment loss vs. HbA1c	0.62	< 0.001*
Clinical attachment loss vs. postoperative glucose	0.67	< 0.001*

## Discussion

This retrospective cohort study evaluated the association between periodontal health and perioperative glycemic outcomes in diabetic patients undergoing dental procedures under general anesthesia. These findings demonstrated that patients with severe periodontitis exhibited significantly poorer perioperative glycemic control than those with mild periodontal disease. Higher preoperative, intraoperative, and postoperative blood glucose levels; increased glycemic variability; a greater incidence of perioperative hyperglycemia; and higher rates of postoperative complications were consistently observed among patients with severe periodontal disease. Furthermore, significant positive correlations between periodontal destruction and glycemic parameters suggest that periodontal status may serve as an important determinant of perioperative metabolic stability in patients with diabetes [4].

The significantly increased HbA1c levels seen in patients with severe periodontitis were one of the study's most notable findings. The well-established reciprocal link between diabetes mellitus and periodontal disease is supported by this biologically plausible conclusion [4]. Through the continuous release of inflammatory mediators including tumor necrosis factor-α, interleukin-1β, and interleukin-6, which disrupt insulin signaling pathways and hinder glucose consumption, chronic periodontal inflammation contributes to systemic insulin resistance [5,6]. Conversely, chronic hyperglycemia enhances the formation of advanced glycation end products, promotes oxidative stress, alters neutrophil function, and accelerates periodontal tissue destruction, thereby creating a vicious cycle of worsening periodontal disease and impaired metabolic control [12]. Similar observations have been reported in previous clinical studies by Taylor [13], Preshaw et al. [14], and Chapple and Genco [15], who demonstrated that severe periodontitis is consistently associated with poor glycemic control and elevated HbA1c levels in individuals with diabetes.

Periodontal bacteria contribute to insulin resistance by inducing chronic systemic inflammation, oxidative stress, and dysregulation of insulin signaling pathways through the release of bacterial virulence factors and pro-inflammatory mediators. These mechanisms impair glucose metabolism, promote hyperglycemia, further strengthen the bidirectional relationship between periodontitis and type 2 diabetes, and have been shown to improve insulin sensitivity and reduce systemic inflammation [16]. The current study also showed that patients with severe periodontitis had significantly higher perioperative blood glucose concentrations at every stage of surgery. The hypothalamic-pituitary-adrenal axis and sympathetic nervous system are activated during surgical procedures performed under general anesthesia, which increases the release of cortisol, catecholamines, glucagon, and growth hormones [17]. These stress hormones enhance hepatic gluconeogenesis and reduce peripheral insulin sensitivity, thereby promoting perioperative hyperglycemia [2,3].

In patients with severe periodontitis, this physiological stress response appears to be amplified by pre-existing systemic inflammation, resulting in exaggerated glucose fluctuations during the perioperative period [18]. Although numerous studies have established a bidirectional relationship between diabetes mellitus and periodontitis and demonstrated that periodontal therapy can improve long-term glycemic control [4,5,13,14,19], the potential influence of periodontal disease severity on perioperative glycemic control during dental procedures under general anesthesia has not been directly investigated. Consequently, evidence regarding the contribution of periodontal status to perioperative glucose fluctuations and postoperative outcomes in patients with diabetes remains limited. Therefore, the present findings provide clinically relevant evidence that supports the importance of periodontal status in perioperative metabolic management.

An additional noteworthy finding was the significantly greater glycemic variability observed in patients with severe periodontitis. Increasing evidence suggests that glycemic variability may contribute more substantially to oxidative stress and endothelial dysfunction than sustained hyperglycemia alone. Large glucose fluctuations promote excessive production of reactive oxygen species, impair endothelial nitric oxide synthesis, and enhance inflammatory cytokine production, thereby increasing the risk of postoperative complications [20]. Dissemination of periodontal pathogens and their virulence factors into the bloodstream contributes to immune dysregulation and has been linked to a number of systemic conditions, including diabetes mellitus, cardiovascular disease, rheumatoid arthritis, poor pregnancy outcomes, and cancer [21]. Periodontitis is a chronic inflammatory disease that destroys tooth-supporting tissues and spreads beyond the oral cavity by raising the inflammatory burden of inflammation. The significantly higher frequency of perioperative hyperglycemia observed in the severe periodontitis group further emphasized the potential contribution of chronic periodontal inflammation to impaired metabolic homeostasis during surgical procedures.

The postoperative outcomes observed in this study further reinforced the clinical significance of periodontal health. Patients with severe periodontitis experience markedly higher rates of surgical site infection, delayed wound healing, and prolonged hospital stay [22]. Hyperglycemia adversely affects wound repair by impairing neutrophil chemotaxis, reducing phagocytic activity, decreasing collagen synthesis, compromising angiogenesis, and delaying epithelial regeneration [23]. Periodontal disease may further increase systemic inflammatory burden, thereby predisposing patients to postoperative infectious complications [21]. These findings are consistent with those of previous reports demonstrating that poor glycemic control is associated with delayed wound healing and increased postoperative infections in patients [24-26]. Similarly, studies by Latham et al. [24], Ata et al. [25], and Martin et al. [26] reported that perioperative hyperglycemia significantly increased the incidence of postoperative infections and prolonged hospitalization across multiple surgical specialties.

Correlation analysis in the present study demonstrated moderate-to-strong positive associations between PPD, CAL, HbA1c, and perioperative glucose levels. These findings suggest that progressive periodontal destruction parallels deterioration of systemic metabolic control. Similar correlations have been reported in several studies that evaluated the relationship between periodontal severity and diabetic status [13,14,16,19]. The consistency of these associations supports the hypothesis that periodontal inflammation should not be regarded merely as a localized oral disease but rather as an important contributor to systemic inflammatory and metabolic dysregulation.

Clinical implications

The results of this study have significant therapeutic ramifications for the multidisciplinary treatment of diabetic patients having general anesthesia for dental procedures. Routine preoperative periodontal assessment may help identify patients at increased risk of perioperative hyperglycemia and postoperative complications. Collaboration among dentists, periodontists, anesthesiologists, oral surgeons, and physicians may facilitate the optimization of both periodontal health and glycemic control before elective surgical procedures. Incorporating periodontal evaluation into preanesthetic assessment protocols may contribute to improved perioperative metabolic stability, reduced postoperative morbidity, and enhanced patient outcomes.

Limitations

The current study had a number of shortcomings. First, a causal relationship between periodontal disease and perioperative glycemic outcomes cannot be established because of the retrospective observational design. Second, the study was conducted at a single institution with a relatively modest sample size, which may limit the generalizability of the findings. Third, residual confounding factors, such as dietary habits, oral hygiene practices, socioeconomic status, and long-term adherence to diabetes treatment, could not be completely controlled. Because periodontal and HbA1c assessments were permitted within a three-month preoperative window rather than being obtained immediately before surgery, temporal changes in periodontal or glycemic status may have introduced some measurement variability. In addition, the evaluation of multiple secondary outcomes and correlation analyses may have increased the risk of type I error due to multiple hypothesis testing. Although Bonferroni correction was applied to post-hoc pairwise comparisons, no formal adjustment was made across all secondary outcomes; therefore, these findings should be interpreted cautiously. Finally, changes in periodontal status following treatment and their influence on perioperative glycemic control were not evaluated. Future prospective multicenter studies with larger populations and interventional designs are warranted to determine whether preoperative periodontal therapy can improve perioperative metabolic outcomes in patients with diabetes.

## Conclusions

Within the limitations of this retrospective cohort study, severe periodontitis was significantly associated with poorer perioperative glycemic control, greater glycemic variability, increased incidence of perioperative hyperglycemia, and higher rates of postoperative complications in patients with diabetes undergoing dental procedures under general anesthesia. The positive correlations between periodontal disease severity and metabolic parameters indicate an association between periodontal status and perioperative glycemic outcomes. Comprehensive periodontal assessment and appropriate periodontal management should be considered integral components of the preoperative evaluation of patients with diabetes to optimize surgical outcomes and reduce postoperative complications.
